# Microphysiological Systems (“Organs‐on‐Chips”) for Drug Efficacy and Toxicity Testing

**DOI:** 10.1111/cts.12444

**Published:** 2017-03-07

**Authors:** LA Low, DA Tagle

**Affiliations:** ^1^ National Center for Advancing Translational Sciences National Institutes of Health Bethesda Maryland USA

## INTRODUCTION

The aim of the Microphysiological Systems (MPS) for Drug Efficacy and Toxicity Testing program, funded by the National Institutes of Health (NIH), was to address difficulties in drug and therapeutic development by creation of human organ microsystems. This program, now entering its fifth year, has achieved all milestones, and is expected to transform the predictive assessment of safety and effectiveness of promising therapeutics by pharmaceutical and regulatory agencies.

### Background of the program

Drug development is costly and time‐consuming, and often has a high attrition rate. Toxicity and a lack of efficacy account for a large number of drug failures during the preclinical and clinical stages of testing of promising targets, in part due to differences between preclinical *in vivo* models (i.e., rodents) and humans in drug metabolizing enzymes and other physiological systems. To address this pressing issue, in 2010 the NIH and the US Food and Drug Administration (FDA) cofunded the Advancing Regulatory Sciences initiative to spur translational work in the regulatory sciences, funding a team at Harvard Wyss to develop a “Heart‐lung micromachine” that contributed towards the highly publicized “lung‐on‐a‐chip.”^1^ Following from this success, in 2012 the NIH, alongside a parallel program from DARPA, tasked researchers with recreating 3D microfluidic organ systems (“tissue chips”) using human cells that represented the characteristics and function of at least 10 major organ systems, and which were to remain viable in culture conditions for at least a month. The aim of these programs was to develop technologies that would be transformative for fast and effective evaluation of safety and efficacy in candidate therapeutics. The NIH funded 19 research projects through two funding opportunities, supporting research to explore the potential of stem and progenitor cells to differentiate into multiple cell types representing the gut, skin, bone, and brain, among others (RFA‐RM‐12‐001), and to develop organ systems including the cardiopulmonary, female reproductive tract, liver sinusoid, kidney, and blood–brain barrier (RFA‐RM‐11‐022).

The 5‐year programs to develop microphysiological systems (MPS) are now in their final year. The DARPA program funded two teams, and focused heavily on integration of multiple platforms into a “human‐on‐a‐chip,” which will end in mid‐2017. The NIH program included an initial 2‐year phase focusing on development of sustainable cell sources (i.e., human induced pluripotent stem cells, hiPSCs), while the second 3‐year phase is now focusing on validation and functional integration of organ systems, and like the DARPA program will end in mid‐2017. The FDA has provided regulatory guidance for both programs since inception. The MPS Consortium consists of NIH awardees and DARPA performers, plus government staff, and meets biannually in person to update Consortium members on recent progress, to share resources and expertise, and to collaborate in order to address challenges precompetitively. The Consortium meetings recently began including workshops with industry partners from the pharmaceutical and biotechnology fields, in order to gain critical feedback on how to move towards reproducibility, validation, and commercialization of MPS technology—a crucial step in making the technology more widely available for the research and pharmaceutical industries.

### Potential applications of tissue chip technology

One of the main aims of the NIH program was to develop systems that could then be accessible to the wider research community, either as tools for the drug development community, or for basic researchers working on understanding organ physiology. The application of MPS within the pharmaceutical industry could help streamline the drug development pipeline by providing “human relevant” assays for screening of promising drugs, but also would provide new avenues for screening of drugs to avoid failures in preclinical or early clinical trials phases. Additionally, with the capability of these platforms to be populated with patient‐derived primary or iPSC sources, “orphan drugs”—those developed for rare diseases that have not yet been marketed— could be screened and tested in ways never before possible in living rare disease populations, potentially opening avenues of new treatments for a range of rare disorders.^2^

For basic researchers, the potential uses of tissue chips are also wide ranging. The utility of these chips to model 3D organ biology could allow new ways to study stem cell differentiation and maturation in *in vivo‐*like systems, and help advance the science underlying the exciting potential of stem cell therapies and regenerative medicine. Additionally, chips representing different populations (age, gender, race) could lead to modeling of specific demographic variants, for a better understanding of genotypic and phenotypic variation within and between populations. Finally, the most useful application would likely be for disease modeling, enabling researchers to study and understand pathologies and organ–organ interactions in disease states in ways that are not currently possible.

### Successes seen…

The list of organs‐on‐chips is ever‐expanding, but within the MPS Consortium, a wide range of tissues and organ systems have been modeled, including the liver sinusoid to mimic hepatic function,^3^ the kidney proximal tubule,^4^ the blood–brain barrier in a “neurovascular unit,”^5^ tissue‐engineered blood vessels,^6^ and gut enteroids.^7^ Multiple groups have designed cardiac systems, monitoring cardiomyocyte differentiation, health, and contractility. For example, the team at Harvard developed “muscular thin films” (MTFs) of aligned cardiomyocytes that deflect according to cellular contractility, and have used that system to model the rare Barth syndrome, with iPSC‐derived tissue then subject to gene editing techniques to correct the X‐chromosome‐linked *TAZ* gene mutation.^8^

As the second phase of this program has focused on platform integration, some teams have focused on representation of multiple systems on their platforms, such as skin, heart, liver, and kidney on a microfluidic platform, or multiple female reproductive tissues (ovarian, fallopian, uterus, and cervix) represented within a linked system. Others have focused on integration of platforms developed by other Consortium members. For example, five groups collaborated to examine the functional linkage of gut, liver, kidney, skeletal muscle, and blood–brain barrier platforms, and are working to show that data from these linked systems are consistent with the published clinical data, and could be used for adsorption, distribution, metabolism, and excretion (ADME) profiling of compounds.

The utility of these platforms for toxicity screening and disease modeling is also being highlighted by the Consortium. For example, “neurospheres” of human iPSC‐derived neurons, glia, and pericytes have been developed and used to give RNASeq readouts from compounds of known toxicity, which have then used machine‐learning algorithms to predict with over 90% success the toxicity of unknown compounds.^9^ Other groups have developed models of vascularized tumor tissues^10^ or metastatic cancers in liver tissues. During the program, supplemental funding has also supported development of additional key organs such as fat, retina, testis, bone marrow, and pancreas, and enabled researchers to use established bioengineered platforms to model rare diseases, including Hutchinson–Gilford progeria syndrome, Timothy syndrome, Alpers–Huttenlocher syndrome, and hereditary hemorrhagic telangiectasia (HHD) using patient‐derived iPS cells.

### …and challenges faced

One major challenge faced by the Consortium has been cell sourcing, as primary cells from human tissue can be difficult to obtain. The use of iPSCs is ideal as a renewable cell source that not only can model healthy states but also disease states, but this has proven challenging. Some organ tissues are less amenable to differentiating into the tissues of choice, and the lack of standardized protocols can lead to large heterogeneity in phenotype and maturity of cells. The stem cell community continues to address this issue.

Another challenge is the use of a common media for physical coupling of platforms. Finding a “blood mimetic” has also been addressed by supplemental funding within the Consortium, as well as by forging close ties with industry to help bring in their expertise. Other challenges have included investigating ways to compensate for the highly lipophilic material PDMS (polydimethylsiloxane), of which many platforms are made due to its pliable and translucent properties, but to which high concentrations of compounds can be lost due to absorption; and how to connect platforms once cells are mature to retain sterility and prevent bubble formation (a big problem in microfluidic devices).

### Future of the program

Organs‐on‐chip technology has the potential to be truly transformative. However, portability and reproducibility of the developed systems must be proven before assay validation can commence and stakeholders can begin to move towards the aim of making the technology more widely accessible. To this end, NCATS recently funded two independent “Tissue Chip Testing Centers” and a Database Center to begin addressing this aim, and have solicited extensive input and expertise from the pharmaceutical industry through the IQ Consortium and the FDA for this effort.

Another initiative that promises to highlight the potential utility of these platforms consists of a partnership between NCATS and the Center for Advancement of Science in Space (CASIS), who will fund four to five teams to adapt their platforms for launch to the International Space Station US National Laboratory (ISS‐NL), in order to use these chips for disease modeling and translational research to benefit human health on Earth and in space.

To further encourage MPS developers to partner with clinical translational efforts, and increase adoption of MPS technology by academic researchers, MPS systems were also listed as areas of high priority under program announcement PAR‐15‐172 for Collaborative Innovation Awards of the NIH's Clinical and Translational Sciences Awards (CTSA)—programs that support a national network of research “hubs” aiming to improve the translational and clinical research process.

Finally, to support continued development and application of the technology, as the first 5‐year program ends, a new 5‐year initiative focused on disease modeling and efficacy testing will begin in July 2017, supported by at least 10 NIH Institutes and Centers. The focus of this program will be to further develop and improve upon established systems to model disease pathologies from patient‐derived iPSCs or genome editing techniques, then using the platforms for therapeutic efficacy testing. The future of Tissue Chip technology is promising, and researchers, industry, clinicians, and patient groups await further advances with interest.
